# Proceedings of the Ohio Dental College Association

**Published:** 1878-06

**Authors:** 


### Proceedings of Societies.

### PROCEEDINGS OF THE OHIO DENTAL COLLEGE ASSOCIATION.

The Ohio Dental College Association met pursuant to adjournment March 5th, 1878, with President J. K. Jamison in the chair.

The amendment to the constitution proposed at the last annual meeting, that nine members shall constitute a quorum was adopted.

Drs. J. S. Rice, A. F. Eminger, D. W. Clancey and J. T. McMillan having fulfilled the requirements were duly elected members of the college association.

Drs. J. Taft, J. I. Taylor and G. W. Keely were appointed a committe to report resolutions upon the death of Dr. B. D. Wheeler, a member of the association.

The treasurer submitted his report showing a balance in the treasury of $309.02, after having paid all indebtedness of the college association.

Dr. H. A. Smith, the secretary, stated that the college property had been removed from the tax duplicate and would hereafter be free from taxation of any kind.

Congratulatory remarks upon the status of the association, in view of the fact that it was free from debt, were made by Drs. Taylor, Taft, Keely, Hunter, Leslie, Smith, Reid and Clayton.

Drs. Jas. Taylor, H. A. Smith, Jas. Leslie, A. O. Rawles and G. W. Keely were appointed a committee to report upon the future management of the Ohio Dental College.

The committee submitted the following:

"Your committee appointed to report on 'what change or improvement they deem necessary in the more vigorous conducting of the Ohio College of Dental Surgery,' beg leave to report as follows: First, we desire to express the great pleasure realized and the sense of relief experienced at the annonncement made by our treasurer that the Ohio Dental

College is now entirely free from the debt that we have been compelled to carry for over twenty years. The alumni scattered all over the world will rejoice that their alma mater has at last freed herself from the pecuniary burden which to some extent has crippled her usefulness. Through these many years she has been sustained by the faithful and unrewarded labors of eminent practitioners, whose love for the college is as keen as in their younger days, but now feel that time lays her hand heavily upon them, and that the valuable trust they have matured so long must be conferred to younger men devoted to this specialty of medicine—second to none in the healing art.

"We have not time to give in detail the improvements that may be thought of, as we know from experience that much in the working details of an institution of this character must be worked up and out by your board of trustees, and we now submit the following as some changes we think worthy of consideration:

"First, that the building be used for no other purpose but dental education, unless by consent of the trustees.

"Second, that the faculty have the use of the building during the winter session; that the summer session be abandoned, and that the dental infirmary open and close with the session.

"Third, that the faculty pay two hundred and fifty dollars per annum into the hands of the trustees, to be used in keeping building, appliances and apparatus in repair.

"Fourth, that the trustees appoint some person to keep a record of all apparatus, books, specimens or other property belonging or donated to the college, and keep them in order for the use of the same.

"The institution now being out of debt, the propriety of reducing the fees for instruction in the college is worthy of serious consideration.

"Drs. Jas. I. Taylor, H. A. Smith, James Leslie, J. R. Clayton, A. O. Rawles and Geo. Keely, Committee."

This was on motion adopted.

The committee to report resolutions expressive of the feelings of the members of this body upon the death of Dr. Wheeler presented the following:

*Whereas*, Since the last annual meeting of this association, our all wise Father has in His providence taken to Himself one of our number, Dr. B. D. Wheeler, who was an active, earnest worker in the cause of professional progress; and especially on subject of dental education did he exhibit a warm interest, and was ever willing to put forth his best efforts for its advancement,

*Resolved*, That in his removal, not only have the profession, society and his family been greatly bereaved, but this association and the institution which it represents, have lost a true and earnest supporter, one who was ever quick to recognize its needs and prompt to supply its wants to the extent of his ability.

*Resolved*, That while we hold pleasant remembrance of that which he did in co-operation with us here, we will not forget his noble qualities as a man, as a member of our profession and of society.

*Resolved*, That these resolutions be received in the proceedings of this body, and that a copy be sent to the family of the deceased.

J. Taft, J. I. Taylor and Geo. W. Keely, committee.

Dr. F. A. Hunter was elected to fill the vacancy in the board of trustees occasioned by the death of Dr. B. D. Wheeler.

Drs. Geo. Watt, J. G. Cameron and A. O. Rawles were elected trustees for three years.

The election of officers resulted in the choice of Dr J. I Taylor, President; Dr. J. F. Canine, Vice-President; Dr. Jas. Leslie, Treasurer; Dr. F. A. Hunter, Secretary.

Adjourned till Tuesday preceeding the first Wednesday in March, 1879.

